# Metaheuristic Optimisation Algorithms for Tuning a Bioinspired Retinal Model †

**DOI:** 10.3390/s19224834

**Published:** 2019-11-06

**Authors:** Rubén Crespo-Cano, Sergio Cuenca-Asensi, Eduardo Fernández, Antonio Martínez-Álvarez

**Affiliations:** 1 Department of Computer Technology, University of Alicante, 03690 Alicante, Spain; rcrespocano@gmail.com (R.C.-C.); sergio@dtic.ua.es (S.C.-A.); 2 Institute of Bioengineering, University Miguel Hernández and CIBER BBN, 03202 Elche (Alicante), Spain; e.fernandez@umh.es

**Keywords:** neural prosthesis, retinal modelling, neural coding, population-based metaheuristic, evolutionary computation, swarm intelligence, multi-objective optimisation

## Abstract

A significant challenge in neuroscience is understanding how visual information is encoded in the retina. Such knowledge is extremely important for the purpose of designing bioinspired sensors and artificial retinal systems that will, in so far as may be possible, be capable of mimicking vertebrate retinal behaviour. In this study, we report the tuning of a reliable computational bioinspired retinal model with various algorithms to improve the mimicry of the model. Its main contribution is two-fold. First, given the multi-objective nature of the problem, an automatic multi-objective optimisation strategy is proposed through the use of four biological-based metrics, which are used to adjust the retinal model for accurate prediction of retinal ganglion cell responses. Second, a subset of population-based search heuristics—genetic algorithms (SPEA2, NSGA-II and NSGA-III), particle swarm optimisation (PSO) and differential evolution (DE)—are explored to identify the best algorithm for fine-tuning the retinal model, by comparing performance across a hypervolume metric. Nonparametric statistical tests are used to perform a rigorous comparison between all the metaheuristics. The best results were achieved with the PSO algorithm on the basis of the largest hypervolume that was achieved, well-distributed elements and high numbers on the Pareto front.

## 1. Introduction

In 2010, the World Health Organisation estimated that the number of blind people in the world amounted to ~40 million [1]. Blindness resulting from retinal infection often leaves the optic nerve and the inner retina relatively intact. Several promising approaches have emerged to restore the sight of patients suffering from these conditions, including gene and stem cell therapies. There is, moreover, one novel technique, the “bionic eye”, which stimulates the visual pathways with an electronic prosthesis [2].

The development of prosthetic systems to restore sight, such as bioinspired sensors, has included interfaces with various visual pathways, resulting in different sorts of visual prosthetic interfaces with the retina, the optic nerve, the lateral geniculate nucleus, the geniculocalcarine tract and the visual cortex. Neural activity is electronically stimulated at an interface along the visual pathway, from where it is relayed to the visual cortex. Leaving the target location aside for one moment, the typical response to electronic stimulation is the elicitation of light percepts—called phosphenes—which generate rudimentary greyscale light perception that gives the patient a visual map for the performance of simple visual tasks and ambulatory movements around fixed objects [3,4]. Unfortunately, visual prostheses are only able to restore a very limited vision with low spatial resolution [5].

Understanding the visual information systems that encode information in the retina is essential for the development of cortical neuroprostheses that can mimic those systems for blind people. The artificially generated signals should be as similar as possible to the signals from the biological retina. Synthetic signals have to be unequivocal and speedy to reproduce the activity of a restricted number of retinal ganglion cells (RGCs), which send processed visual information through the optic nerve to higher visual centres. Considering those points, we have extended a highly parametrised bioinspired retinal model framework, first presented in [6], to explore new tuning possibilities. This bioinspired mathematical model describes the different stages that comprise the vertebrate retina, simplifying its behaviour to generate a low latency model with high throughput in real-time. In the first stage, the antagonist central–peripherical behaviour of the receptive ganglion cells are modelled and, in the second stage, the ganglion cell firing behaviour is reproduced. All parts of the mathematical model have many parametrical candidates to be adjusted, most of which are real numbers with, in reality, an infinite range of possible values. Therefore, a straightforward understanding of the fine-tuning of those parameters represents a difficult problem that is addressed through an extension of our previous work [6,7], in which an automatic population-based multi-objective strategy was proposed to optimise model performance. We now propose a strategy that consists of applying metaheuristic optimisation algorithms to fit the model together with analytical tools for evaluating and comparing the results of each metaheuristic process.

The central contribution of this study is its comparison of different metaheuristic optimisation algorithms for fine-tuning a bioinspired retinal model of responses to artificial stimuli in mice retinas. In our study, a retinal model is optimised using four objective functions, which not only describe relevant features of the response, such as the areas of each receptive field, but also improve the spike time predictions. In this way, the recurrent problem of oversimplified parametric model fitting is surmounted, by addressing a multi-objective complex problem. The computational retina is modelled on the collective responses of a set of retinal ganglion cells, the characteristics of which mean that the mathematical model is capable of high functional performance—low latency and high throughput—in real-time. The use of biological extracellular recordings from mice provided more precise models. As far as is known, this study reports, for the first time, the evaluation of population-based multi-objective metaheuristics in relation to the fine-tuning of a retinal model. Our work, therefore, attempts to demonstrate the feasibility of applying population-based metaheuristics to fine-tune a retinal model in real-time. Research into highly parametric frameworks is of immense value for the development of prosthetic devices that will be capable of restoring functional sight to many blind people through direct stimulation of the visual cortex. This study is therefore important, because it responds to the challenge of designing an intracortical visual neuroprosthesis that can dialogue with the occipital visual cortex, as a means of transferring limited, but useful, visual data to patients who would otherwise remain blind [8,9].

Thus, the central objective of this work is to demonstrate that an automatic optimisation strategy, capable of optimising a bioinspired retinal model that can function in real-time, is feasible by means of metaheuristic optimisation algorithms. The novelty of this work involves an optimisation strategy that enables the adjustment of the retinal model, to predict retinal ganglion cell responses. Four biologically based metrics (peristimulus time histogram, interspike interval histogram, firing rates and neuronal receptive field size) were selected as objective functions for comparing and evaluating the different individuals that form the population of candidate solutions to the problem. Unlike traditional retinal adjustment methods where only the explained variance or correlation coefficient is optimized (a single objective), our approach optimises the bioinspired retinal model by using those four metrics which are obtained from wild type mice retinal ganglion cell recordings. Five different search heuristics were proposed for fine-tuning the parameters of the retinal model in a scientific analysis that has not been previously conducted. Finally, nonparametric statistical tests were used to perform a rigorous comparison between all the metaheuristic models, providing a robust and innovative optimisation strategy. In summary, a multi-objective retinal model optimisation strategy has, for the first time, been presented that performs a comparison between different metaheuristic strategies using nonparametric statistical methods.

In the remainder of this paper, the background to this research will be presented in Section 2. The materials and the methods used in this study will be described in Section 3. In Section 4, the experimental results will be reported, and, finally, the concluding comments will be presented in Section 5.

## 2. Related Work

**Figure 1:**
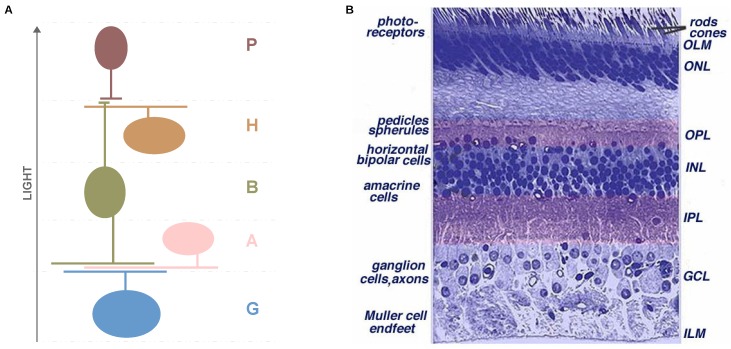
Anatomy of the retina. (**A**) Schematic diagram of the connections between the basic cell classes—photoreceptors (P), horizontal cells (H), bipolar cells (B), amacrine cells (A) and ganglion cells (G). Light rays must reach the sensory cells, the photoreceptors, passing through the entire retina. (**B**) Light micrograph of a vertical section of the central human retina (adapted from Webvision [19]).

Over the last 100 years, vertebrate retinas have been the object of detailed scientific study and analysis throughout the world [10,11,12,13,14,15,16,17]. Pioneering studies on the basis of neuroanatomy for the vertebrate nervous system, in general, and anatomic cellular descriptions of the retina, in particular, were proposed by Ramón y Cajal in 1892 [18]. Vertebrate retinas are mainly composed of three very thin layers of nerve cell bodies and two layers of synapses. The Outer Nuclear Layer (ONL) consists of a large number of photoreceptor cells—rod cells and cone cells; the Inner Nuclear Layer (INL) consists of the horizontal cells, bipolar cells and amacrine cells; and the Ganglion Cell Layer (GCL) consists of ganglion cells and displaced amacrine cells. Figure 1A shows a simplified schematic diagram of the connections between the basic cell classes of the retina and the pathways for light to reach the photoreceptor layer. There are two layers of neuropils between those three layers where synaptic contacts are regulated. The first neuropil layer is the Outer Plexiform Layer (OPL), consisting of a dense network of synapses between photoreceptor cells and horizontal cells. The second neuropil layer is the Inner Plexiform Layer (IPL), consisting of synaptic connections between bipolar cells and ganglion cells [19]. Figure 1B reveals the complexity of human retina in a vertical section.

Several computational models have been proposed to imitate the behaviour of the vertebrate retina from the single cell to the network level. Simoncelli et al. proposed a method for the characterisation of the functional relationship between environmental stimuli and their neural responses, using the concept of the receptive field. This model combines a linear spatiotemporal filter of single static nonlinearity [20]. Paninski et al. proposed statistical model-based techniques that provide a unified solution to the encoding problem (how information is encoded in neural spike trains) and the decoding problem (how much information is encoded in a spike train) [21]. Pillow et al. analysed the functional significance of correlated firing in a complete population of macaque parasol retinal ganglion cells using a multi-neuron spike response model [22]. Nirenberg et al. presented a data-driven model of retinal input–output relationships that is effective on a broad range of stimuli [23,24,25]. This prosthetic system converts visual input into the same patterns of action potentials that the retina normally produces. It reliably reproduces those patterns for a broad range of stimuli, including faces, landscapes and animals. Wohrer and Kornprobst proposed a computational simulation software, called Virtual Retina, which performs large-scale simulations of high biological plausibility [26]. Cessac et al. extended the range of retina simulation software with a new platform that integrates the retina simulator and a toolbox for the analysis of spike train population statistics [27]. Huth et al. published Convis, a Python simulation toolbox for large-scale neural populations, which offers luminance gain control, contrast gain control and arbitrary receptive fields by 3D convolutions executed on a graphics card [28]. Martínez-Álvarez et al. proposed a compiler-based framework capable of describing, simulating and validating customized retina models [29]. Martínez-Cañada et al. proposed a set of computational retinal microcircuits that can be used as basic building blocks for the modelling of different retina mechanisms [30]. Recently, deep convolutional neural networks were shown to capture retinal responses to natural scenes, with results that were close to the variability of the cellular response range [31,32], as well as multitask recurrent neural networks that provided the necessary flexibility to model complex neuronal computations [33].

Currently, there are several research groups working towards the development of visual prostheses, among which may be highlighted the Argus II epiretinal implant from Second Sight Medical Products Inc. [34,35], the sub-retinal visual implant Alpha IMS/AMS from Retina Implant AG [36,37], the Boston Retinal Implant [38], the IRIS II and PRIMA devices from Pixium Vision Inc. [39] and Epi-Ret 3 [40]. In addition to the aforementioned retinal prostheses, other research groups are also working on the development of cortical visual prostheses [9,41,42].

## 3. Materials and Methods

### 3.1. Retinal Model under Study

Most retinal models are normally described using hierarchical processing models, which are influenced by the biological plausibility of vertebrate retinas. Photoreceptor cells detect visible light within the retina, where horizontal, amacrine and bipolar cells process the signals, and retinal ganglion cells that integrate their output in the form of action potentials that are relayed to the higher visual centres. Retinal neurons, in contrast, form centre-surround receptive fields that react to spatial changes.

**Figure 2:**
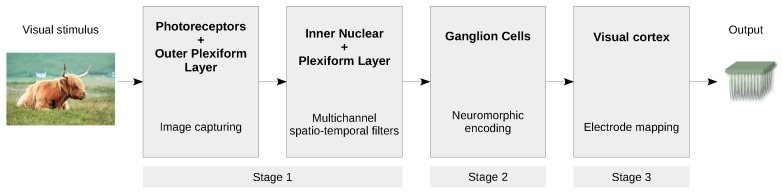
Flowchart of the bioinspired retinal model (BIRM) under study. Stage 1 is modelled as a weighted sum of several well-known convolutive spatiotemporal image filters, such as Difference-of-Gaussian (DoG) and Laplacian-of Gaussian (LoG) filters. Stage 2 is modelled by the noisy leaky integrate-and-fire model (NLIF). Stage 3, which represents the potential electrode remapping, is beyond the scope of this paper. This figure is a derivative of “Highland cattle, Skye Island” by Qu1m, used under CC BY, and Webvision [19].

Figure 2 shows the basic processing stages of the bioinspired retinal model (BIRM) under study. In the first stage (Equation (1)), a weighted sum of several spatiotemporal filters over the captured stimulus is performed; then, in the second stage, the neuromorphic encoding (Equation (2)) is accomplished; finally, the electrode mapping that addresses the output from the second stage to the corresponding electrodes takes place in the third stage.

The difference-of-Gaussian (DoG) model was used to implement the first stage of the model, to imitate the spatial centre-surround opposition performed by retinal neurons. The use of image filtering using the DoG technique, to imitate the centre-surround RGCs, was proposed by Rodieck [43] and Enroth-Cugell [44]. Furthermore, the Laplacian-of-Gaussian (LoG) model proposed by Marr and Hildreth [45] was used to capture the “Mexican hat” shape—with large values in the centre and small opposite polarity values in the surround—of the receptive fields of RGCs. The combination of both those models—DoG and LoG—formed the first stage of the model—BIRM, computed with the following equation,

(1) $$\begin{array}{c} S_{1}=W_{1}\cdot f_{DoG}\left(\sigma_{1},\sigma_{2},\mu_{1},\mu_{2},k_{1},k_{2},R+B,G\right)\\ +W_{2}\cdot f_{DoG}\left(\sigma_{1},\sigma_{2},\mu_{1},\mu_{2},k_{1},k_{2},R+G,B\right)\\ +W_{3}\cdot f_{LoG}\left(\sigma_{1},\sigma_{2},\mu_{1},\mu_{2},k_{1},k_{2},I\right) \end{array}$$

where DoG is the difference-of-Gaussian filter; LoG is the Laplacian-of-Gaussian filter; σ is the parameter of the Gaussian filter; μ is the parameter of the Gaussian filter; *k* is the kernel size of the Gaussian filter; *R* is the red channel; *G* is the green channel; *B* is the blue channel; *I* is the intensity channel; and $W_{1}$, $W_{2}$ and $W_{3}$ are weight variables.

Neuromorphic encoding is performed in the second stage of the model through the noisy leaky integrate-and-fire (NLIF) model [46,47,48]. This model describes neurons as simple electrical circuits consisting of a capacitor in parallel with a resistor driven by a noisy input. The noise current consists of Gaussian white noise and represents the net contribution of all noise sources to the membrane potential. This neuromorphic encoding model is computed by the following equation,

(2) $$\begin{array}{c} S_{2}=NLIF\left(S_{1},t,l,rp,pt,fmf\right) \end{array}$$

where $S_{1}$ is the output of the Equation (1), *t* is the threshold parameter, *l* is the leaky parameter, rp is the refractory period parameter, pt is the persistence time parameter and fmf is the frequency modulation factor. When the accumulated membrane potential exceeds the threshold value, *t*, an action potential is generated, and the accumulated membrane potential is then reset to 0 for a time, rp. The parameter *l* modulates the diffusion of ions through the cell membrane, the parameter pt modulates the number of times the image is processed—to move ever closer to the continuous processing of the retina—and the parameter fmf modulates the shape of the transient response of the RGC.

Every retinal model (BIRM) is encoded by a set of mixed integer and floating-point values (x∈R). Given that there are a series of restrictions associated with each parameter, each solution to the optimisation problem must satisfy each of those restrictions, to represent a valid strategy. As a consequence, whenever a new instance of the BIRM is generated, each module in charge of generating each parameter must comply with all the restrictions.

### 3.2. Human Visual System Modelling

Benoit et al. proposed an image processing approach to copy the Human Visual System (HVS) by modelling some of its parts, to develop low-level image processing modules [49]. The study showed the advantages of using such modelling, to develop efficient and rapid human vision inspired modules for low-level image processing.

**Figure 3:**
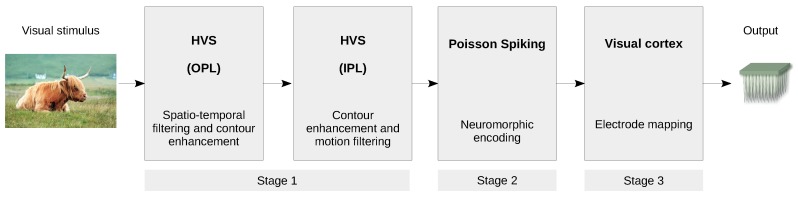
Functional processing blocks of the Human Visual System Poisson (HVSP) model. This figure is a derivative of “Highland cattle, Skye Island” by Qu1m, used under CC BY, and Webvision [19].

This image processing approach was used to model the input–output relationships of the retina, to provide a baseline for comparison. The model consisted of a cascade of a first stage with the spatiotemporal filtering of the HVS model, followed by a Poisson spike generator (HVSP) (see Figure 3). Poisson processes cannot mimic certain retina properties [22], i.e., bursting or refractoriness, though they can be used as a baseline for comparison, due to their computational simplicity.

### 3.3. Metaheuristic Optimisation Algorithms

**Figure 4:**
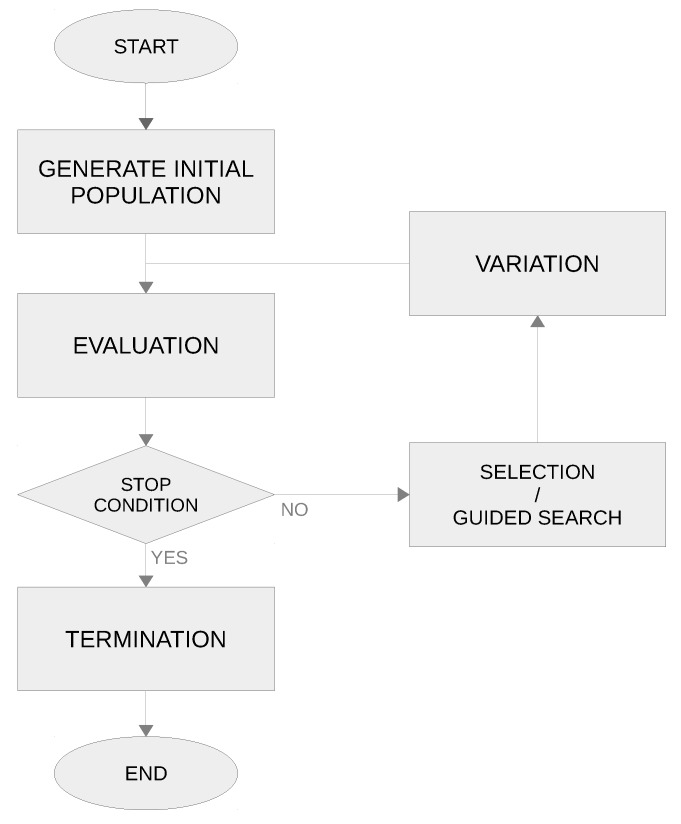
Basic flow chart of a general population-based metaheuristics.

Multi-objective optimisation is simultaneously intended to optimise mathematical optimisation problems with more than one objective for which no single solution exists. Those optimisation problems are called multi-objective optimisation problems (MOPs). Metaheuristics belong to the family of stochastic optimisation methods where nature-inspired mechanisms (based on some principles from ethology or biology) are imitated for solving optimisation problems [50]. Traditionally, metaheuristics have been classified as single-solution based metaheuristics and population-based metaheuristics in the literature. Single-solution metaheuristics deal with a single initial solution, whereas population-based metaheuristics deal with a population of candidate solutions. The most widely used population-based methods are Evolutionary Computation (EC) and Swarm Intelligence (SI). Inspired by Darwin’s Theory of Evolution [51], EC algorithms are based on the principles of natural selection, where inherited genetic variations help organisms survive, reproduce and compete. Among the most prominent algorithms are genetic algorithms [52,53], genetic programming [54], evolution strategies [55] and cultural algorithms [56]. On the other hand, SI algorithms are inspired from the collective behaviour of animal and insect societies, where a population of individuals/agents interact in a very limited way with their environment, performing complex tasks, so as to ensure their survival. The main metaheuristics belonging to this field are particle swarm optimisation [57], bee colony optimisation [58], ant colony optimisation [59] and bacterial foraging optimisation [60]. Figure 4 shows the basic flow diagram of population-based optimisation metaheuristics.

In addition, metaheuristic optimisation algorithms have been validated in other areas of knowledge, such as industrial soft sensor modelling [61], wireless sensor networks [62,63,64,65,66], privacy-preserving data mining [67] and RFID network planning [68,69]. A brief description of main population-based optimisation metaheuristics is summarised below.

#### 3.3.1. Genetic Algorithms

Genetic Algorithms (GA) are a set of population-based search heuristics inspired by the natural selection process by means of three genetic operators, i.e., mutation, crossover and selection [52,53]. The population of candidate solutions is formed by chromosomes and the best are carefully chosen as ascendants that will create new descendants. Individuals are selected by means of a fitness-based process, where fitter solutions are selected with higher probabilities. Finally, the mutation operator is used, to maintain population diversity and to prevent premature convergence. A description of the adaptation of the GA algorithm is shown in Algorithm 1.

Deb et al. [70] presented the nondominated sorting genetic algorithm II (NSGA-II) as a Pareto-based ranking scheme. The algorithm searches for nondominated solutions by classifying, in terms of Pareto dominance, the population of individuals at different levels. Furthermore, the crowding distance metric is used to generate an estimation of the density of the solutions surrounding any particular one in the population. Solutions with smaller values are more crowded, so higher crowding distance values are preferred. After the mating selection process, parent and offspring populations are combined and the resulting population is truncated deleting the worst 50%.

The Strength Pareto Evolutionary Algorithm (SPEA) proposed by Zitzler and Thiele [71] is a genetic algorithm for finding Pareto-optimal solutions of MOPs. The SPEA algorithm uses an external archive of a predefined size to preserve all the nondominated solutions. Briefly, the individuals are selected from the union of the population and the external archive, and the mating process is performed through binary tournaments, followed by the mutation and recombination stages. Finally, the entire population is replaced by the new descendants. Even though SPEA has a good performance, several studies have identified potential weaknesses, such as fitness assignment, density estimation and archive truncation. SPEA2 was designed to surmount the aforementioned issues [72]. The new features of SPEA2 are the use of a new fitness assignment that incorporates density information, the external archive size is fixed over the time, only the archive members participate in the mating selection process and the new clustering technique retains the boundary points. These characteristics mean that SPEA2 can perform better than its predecessor for all problems. Finally, SPEA2 appears to perform better in higher-dimensional objective spaces than PESA [73] and NSGA-II [70].

Recently, a new procedure for solving multiple-objective optimisation problems—NSGA-III—was proposed by Deb and Jain [74]. Many-objective optimisation problems refer to a group of problems with four or more objectives. This population-based heuristic maintains the population diversity by using a number of well-spread reference points, finding a well-converged and well-diversified set of solutions in all test problems. Whereas mainstream multi-objective evolutionary algorithms may have trouble solving optimisation problems with more than four objectives, the NSGA-III algorithm can produce satisfactory results for problems with up to 15 objectives.


**Algorithm 1: Multi-Objective Genetic Algorithm.**


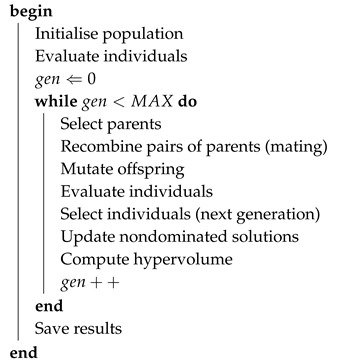



#### 3.3.2. Particle Swarm Optimisation

Based on simplified animal social behaviours and the movement of organisms such as bird flocking or fish schooling, Kennedy [57] proposed a population-based search heuristic and optimisation technique with nonlinear functions called Particle Swarm Optimisation (PSO). PSO is included in the field of optimisation metaheuristics and has been found to be successful in a wide variety of optimisation problems such as image and video analysis applications [75], electromagnetics [76] and scheduling [77].

After a random initialisation of a swarm of candidate solutions, referred herein as particles, the algorithm finds the best global solution by moving the particles in the search space towards its own best position and towards the best particle of the entire swarm on the basis of its position and velocity. Numerous improvements of the initial metaheuristic model were subsequently presented [78,79,80].

For the purpose of solving multi-objective optimisation problems, the original structure of the PSO algorithm needs to be modified. An external archive was used in this study to keep the Pareto-optimal individuals, working as leaders in the swarm particle updating process. The reader interested in alternative approaches can consult the state-of-the-art published by Reyes-Sierra and Coello [81]. A description of the adaptation of the PSO algorithm is shown in Algorithm 2.


**Algorithm 2: Multi-Objective PSO Algorithm.**


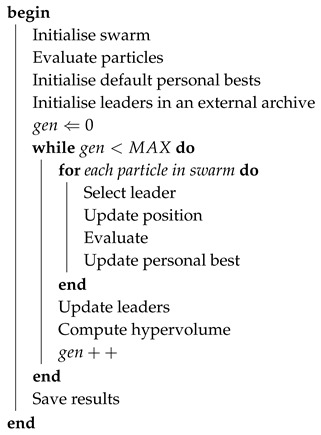



#### 3.3.3. Differential Evolution

Differential Evolution (DE), proposed by Storn and Price [82], is a population-based algorithm that is used to solve optimisation problems. This method optimises a problem by iteratively generating new solutions through recombination and mutation operators. The mutation process is the basic operation, in which a new descendant is produced, based on the combination of the candidate solution with differences between both individuals. The population size is maintained through a replacement procedure where the newly generated individual competes against its corresponding predecessor and replaces it only if it has a better fitness value.

As in the PSO algorithm, the original structure of the DE algorithm needs to be modified for the purpose of solving multi-objective optimisation problems. Robič and Filipič [83] suggested an approach to multi-objective problems named Differential Evolution for Multi-Objective Optimisation. The “*DE/rand/1/bin*” variant was used in this study, where “DE” refers to the name of the algorithm and the rest of the parameters indicate that a pair of solutions are randomly selected. Finally, a binomial recombination is used to build the descendant solution candidate. The methodology to maintain the population size is as follows; if a descendant dominates its predecessor, that individual is replaced; if a descendant is dominated by its predecessor, it is discarded; otherwise, the new descendant is added to the population. After the creation process of the offspring, this algorithm applies a nondominated sorting mechanism combined with the use of the crowding distance measure (derived from the original NSGA-II algorithm), to maintain the population size. A description of the adaptation of the DE algorithm is shown in Algorithm 3.


**Algorithm 3: Multi-Objective DE Algorithm.**


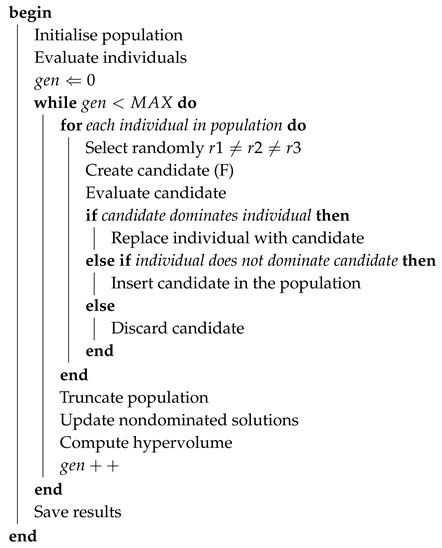



### 3.4. Retina Preparation, Multi-Electrode Recordings and Spike Sorting

Biological records were used to perform acceptable evaluations for the purpose of obtaining fine-tuned models. Extracellular recordings from populations of retinal ganglion cells were obtained from wild type (C57BL/6J strain) mice. Both eyes were removed following anaesthesia with 4% isoflurane inhalational and cervical dislocation. Both the cornea and lens were removed from the eyeball. The retinas were removed from the remaining eyecup with the pigment epithelium and mounted on an agar plate with the ganglion cell side facing up. A piece of nitrocellulose paper covered the tissue for the purpose of maintaining the correct wetness. All experimental procedures were performed in accordance with the ARVO and European Communities Council Directives (86/609/ECC) for the use of laboratory animals. The entire procedure was performed under dim red illumination.

Extracellular recordings were obtained from the ganglion cell layer, by means of an array of 100 electrodes [84]. Electrical signals captured by the array electrodes were digitalized and stored using a data acquisition and signal processor system. Spike sorting was accomplished by well-known clustering algorithms and Principal Component Analysis (PCA).

The visual stimuli covered an area of 120×154 pixels at 60 Hz refresh rate. Stimuli was resized to a 4 mm ×4 mm area by means of optical lenses and projected through a beam splitter focusing the stimulus onto the photoreceptor layer. Several repetitions of a 700 ms flash were displayed followed by darkness for 2300 ms to classify the ganglion cells in ON, OFF and ON/OFF. The biological retinas were then stimulated with 250 μm wide white bars passing across a black screen at 1 Hz in eight orthogonal directions.

**Figure 5:**
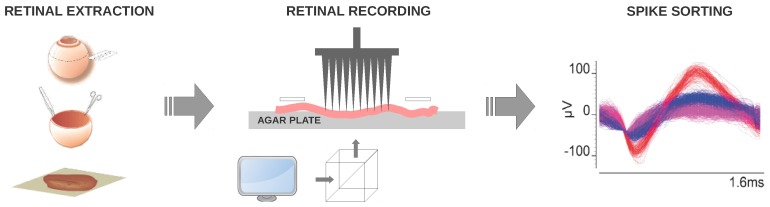
Data acquisition procedure of multi-electrode recordings and spike sorting.

Figure 5 summarises the above procedure for the acquisition of electrophysiological recordings. Readers with an interest in the procedure for obtaining extracellular recordings may consult a detailed description in [6,85].

### 3.5. Objective Functions

Four objective functions were selected for the purpose of comparing biological and synthetic electrophysiological recordings and to check whether a given design solution was close to achieving the set aims. The electrophysiological recordings obtained from the ganglion cells of mice consist of spike train data. Data generated by the retinal framework are likewise comprised of spike train data for all simulated ganglion cells. Kullback–Leibler Divergence (KLD) [86] (also known as relative entropy) was employed to assess the similarity of the PSTH response (PSTH-KLD), so as compare to and contrast with the peristimulus time histograms (PSTH). The interspike interval histogram (ISIH) data of each ganglion cell was also measured with the same technique (ISIH-KLD). For the purpose of measuring the spike count rate, the absolute difference of the firing rate (FRAD) was selected as one of the objective functions. Last, RGCs are known to integrate the signals from a group of afferent neurons, forming the neuron’s receptive field (RF), which comprises a two-dimensional region in a visual space of varying size. The method proposed by Díaz-Tahoces et al. was used for the purpose of estimating the neuron’s receptive field [6,85]; therefore, the last metric selected as one of the objective functions was the absolute difference of the neuron’s receptive field size (RFAD). Those four objective functions—PSTH-KLD, ISIH-KLD, FRAD and RFAD—were selected as objective functions of the proposed framework to guide simulations towards optimal design solutions.

As detailed above, this study addresses a multi-objective optimisation problem in which all objectives should be minimised. Two of the metrics—PSTH-KLD and ISIH-KLD—represent the relative entropy, and are responsible for measuring the similarity between two distributions. Therefore, they should be minimised. Likewise, the FRAD should be minimised, as the firing rate should be as similar as possible. Lastly, models of greater reliability were obtained, by ensuring that the area of the receptive field of the RGCs was of an appropriate and similar size.

### 3.6. Evaluation of Multi-Objective Optimisation Metaheuristics

The goal of multi-objective optimisation is to solve nontrivial multi-objective optimisation problems where no single solution exists. All objective functions are said to be conflicting and have to be optimised simultaneously. Therefore, only a good approximation of the Pareto frontier can be obtained. Furthermore, the results produced by multi-objective optimisation metaheuristics are difficult to compare between each other. The resulting sets of best solutions of distinct population-based search heuristics must be compared to evaluate and compare their performance. All metaheuristics optimise the same model parameters, as the aim is to study the best performing algorithm. One of the most commonly used performance metrics is the hypervolume (HV) indicator [87,88], which is capable of mapping complex solutions with more than two objectives to a single value that can be easily used to compare the solution sets. The hypervolume indicator is also known as the *S* metric or the Lebesgue measure. Briefly, the HV metric represents a calculation of the volume of the dominated area of the objective space. The HV metric may be simply described as the volume of the objective space contained between a previously defined reference point and the Pareto-optimal solutions. Evaluation of multi-objective optimisation metaheuristics is of great interest because they possess the highly beneficial characteristic of strict Pareto compliance [89,90].

Other performance metrics, such as Inverted Generational Distance [91], were rejected, due to the fact that the exact Pareto-optimal sets are unknown. Moreover, the HV metric has the characteristic that the HV value is higher for solution sets that dominate all other sets in relation to Pareto dominance.

### 3.7. Statistical Interpretation

Due to the stochastic nature of the population-based metaheuristics, statistical tools should be used to compare the results of the experiments. The most straightforward strategy for the experimental design is to run each algorithm for a specified number of independent executions and then obtain some descriptive statistics of the results, such as the means and standard deviations. The use of graphs and charts for this task can be useful. Box plots [92,93] are one of such representations, because they allow a visual comparison of the results, from which some conclusions can be drawn.

Given the nature of the problem, as each individual must process a stimulus of considerable duration, running each experiment is computationally expensive. Having analysed the variability of the hypervolume, it was, therefore, decided that each of the experiments (metaheuristics) would be performed 10 times.

Nevertheless, it was also necessary to conduct a set of statistical inferences that would support the conclusions drawn from the data. The use of parametric methods is not advisable, in order to assess and to compare multi-objective optimisation metaheuristics, because the data distributions are unknown. Conversely, nonparametric methods can be used to decide when a stochastic metaheuristic is considered better than any other. Nonparametric methods were therefore selected to analyse the data, in order to follow a methodology for comparing metaheuristic optimisation algorithms. Specifically, the Kruskal–Wallis test [94], the Friedman test [95,96] and the Mann–Whitney–Wilcoxon U test [97,98] were applied in this study. The results must furthermore be adjusted due to multiple comparisons, for which purpose the Bonferroni–Dunn procedure [99], the Hochberg procedure [100] and the Hommel procedure [101] were applied.

## 4. Experimental Evaluation and Results

Biological extracellular recordings were utilised as reference standards for fine-tuning the model under study, BIRM, through five different multi-objective optimisation metaheuristics—SPEA2, NSGA-II, NSGA-III, PSO and DE. Then, a preliminary analysis of all the models was performed. As shown in Section 3.4, mice retinas were stimulated with 250 μm wide white bars set at different angles—$0^{\circ }$, $45^{\circ }$, $90^{\circ }$, $135^{\circ }$, $180^{\circ }$, $225^{\circ }$, $270^{\circ }$ and $315^{\circ }$—passing across a black screen.

All multi-objective optimisation metaheuristics were developed, as stated in the original publications, and all hyperparameters were set as suggested in their original studies. The probabilities of crossover and mutation operators were fixed to 0.40 and 0.05, respectively. All the experiments were initialised with a population of 40 individuals and were simulated over 100 generations. Those parameters were selected following the results of preliminary experiments, to determine the balance between performance and execution time. The parameters of the PSO search heuristics—$\phi_{1}$ and $\phi_{2}$—were both set at 2.05, as stated in the original study. For the DE search heuristic, the parameter *F* was fixed to 1 as stated in the original study. For all multi-objective optimisation metaheuristics, identical population and archive sizes were used. Table 1 summarises the parameters of the retinal model BIRM, which were selected to be automatically changed, together with their range of variations. The kernel sizes of the first stage of the model (see Figure 2) were restricted between 3 and 13, based on a previous manual analysis of the neuron’s receptive fields. In contrast, the parameters of the second stage, NLIF, were identified, as the most important parameters of the retinal model when stimulated with an artificial stimulus (white bars passing across a black screen) and the ranges were determined based on manual tests. Regarding the type of data, two chromosome parameters—PersistenceTime and *K*—were set as integers, and the rest were set as floating-point numbers. All the parameters were selected on the basis of their relevance in relation to the selected stimulus, following a thorough analysis of the constraints and the boundaries. All other parameters of the retinal model were fixed and remained unchanged during the training process (see Equation (1)). Last, as stated in Section 3.5, four objective functions were selected to indicate how close each design solution was to the expected biological extracellular data.

**Figure 6:**
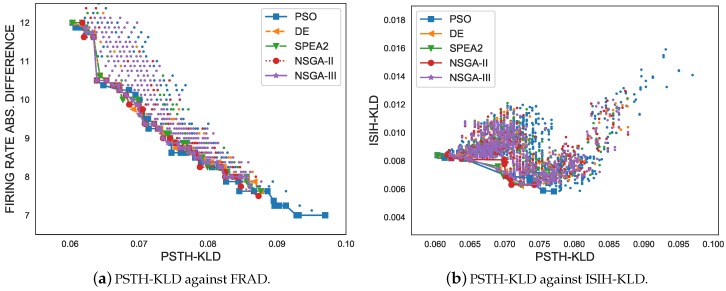

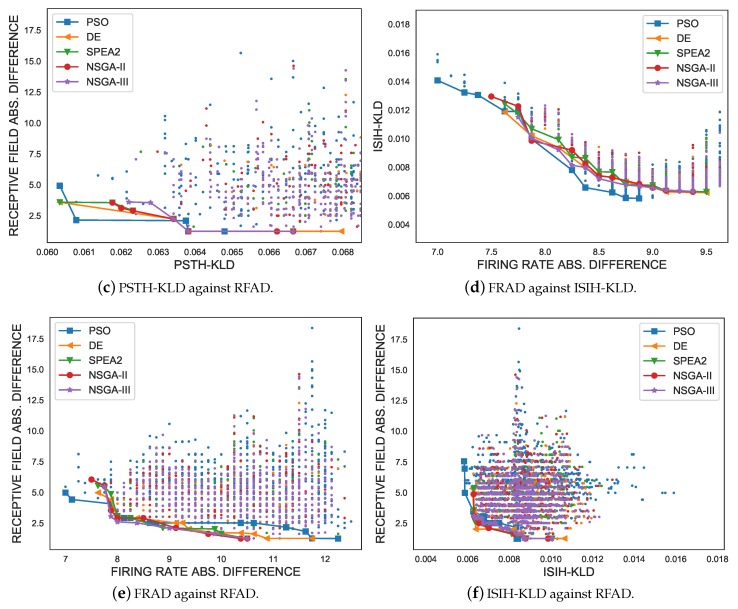
Multi-objective graphs and Pareto-optimal sets of all multi-objective optimisation metaheuristics facing each pair of criteria. (**a**) The PSTH-KLD metric against the Firing Rate Absolute Difference (FRAD) metric with their Pareto fronts; (**b**) The PSTH-KLD metric against the ISIH-KLD metric with their Pareto fronts; (**c**) The PSTH-KLD metric against the RFAD metric with their Pareto fronts; (**d**) The FRAD metric against the ISIH-KLD metric with their Pareto fronts; (**e**) The FRAD metric against the RFAD metric with their Pareto fronts; (**f**) The ISIH-KLD metric against the RFAD metric with their Pareto fronts.

The visualisation of the results and their interpretation was expected to be extremely difficult in all MOPs with more than three objectives: the greater the number of objectives, the more complex the problem and the greater the difficulty for a decision-maker wishing to select a preferred solution. First, as part of the preliminary assessment procedure, Figure 6 shows a set of charts with the results and the Pareto fronts with the objective functions simultaneously minimised for a single experiment. All data were represented by drawing a comparison in pairs for each of the metrics. As can be observed, all the Pareto individuals were nearly identical. However, it cannot be argued from a pairwise comparison of the metrics that one of the metaheuristics is better or worse than all the others. In contrast, the Pareto fronts for the experiments with NSGA-III and PSO have more individuals, which is desirable. For example, in Figure 6a, the Pareto fronts of the SPEA2 algorithm and the NSGA-III algorithm are shown to contain 34 and 46 individuals, respectively, and the PSO algorithm contains 52 individuals. In Figure 6f, the NSGA-III algorithm evidently outperforms the other metaheuristics with 21 individuals. In Figure 6d, the PSO experiment obtained better results than the other metaheuristics, because of its well-spread Pareto front that dominates the rest of Pareto sets. In summary, after a preliminary analysis, the data to determine which of the multi-objective optimisation metaheuristics generated better results were not conclusive, but the PSO and NSGA-III algorithms generated larger-sized Pareto sets.

**Figure 7:**
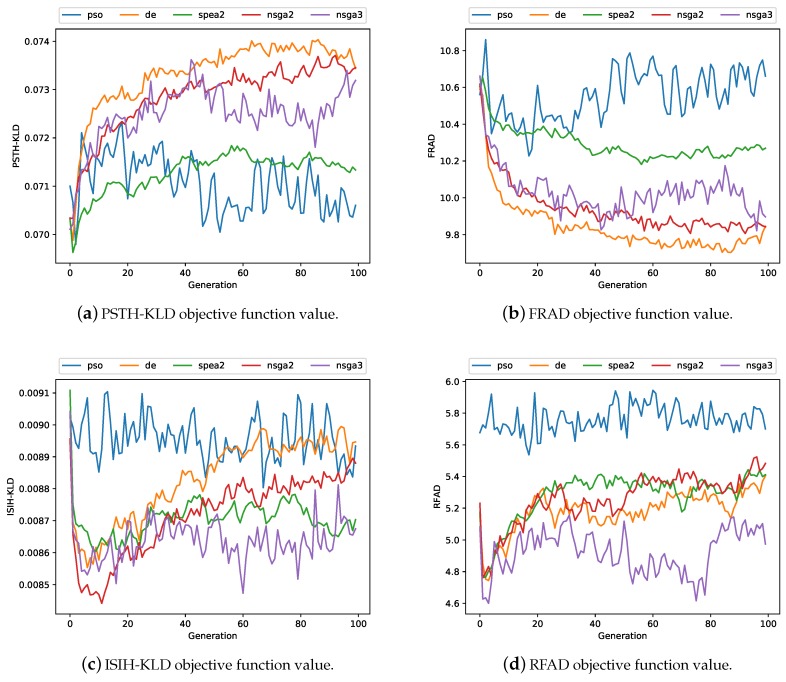
Average value of the objective functions over 100 generations: Particle Swarm Optimisation (PSO) (blue), DE (orange), SPEA2 (green), NSGA-II (red) and NSGA-III (purple).

**Figure 8:**
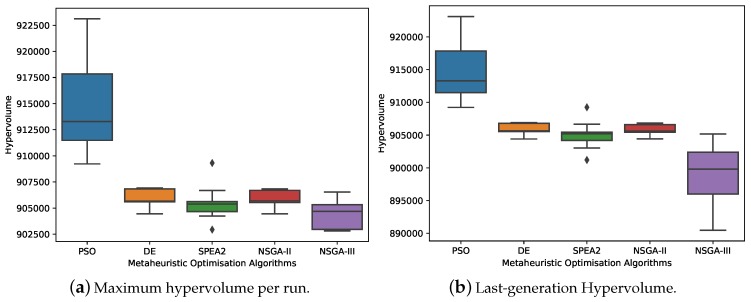
Distribution of the hypervolume metric for 10 independent runs of all multi-objective optimisation metaheuristics.

For the purpose of avoiding the stochastic consequences of all multi-objective optimisation metaheuristics, 10 independent executions were performed per metaheuristic. As indicated in Section 3.7, the experiments are computationally expensive (the computation time for each iteration required was ~20 min), so it was estimated that running each experiment 10 times would avoid stochastic effects. As described in Section 3.6, a metric that can be used to evaluate the results was necessary, due to the multi-objective nature of the problem. Figure 7 shows the average values of the four objective functions for all the executions, from which no clear conclusions can be drawn. As for the hypervolume, Figure 8a shows the distribution of the maximum HV metric of the 10 experimental executions, and Figure 8b shows the distribution of their last generation. The data distribution of the HV metrics is represented below in two box plots, enhancing our understanding of the sample data, which will help us draw comparisons between samples. Whenever one approximation dominates another approximation, the HV of the former will be greater than the HV of the latter. It appears that a variant of the NSGA-II algorithm, as the selection algorithm for the maintenance of the population size in the DE experiments, produced very similar results. In contrast, SPEA2 results were very similar to those for DE and NSGA-II HV, although their distributions were wider. The worst results were obtained with the NSGA-III algorithm and the best results, with the PSO algorithm.

**Figure 9:**
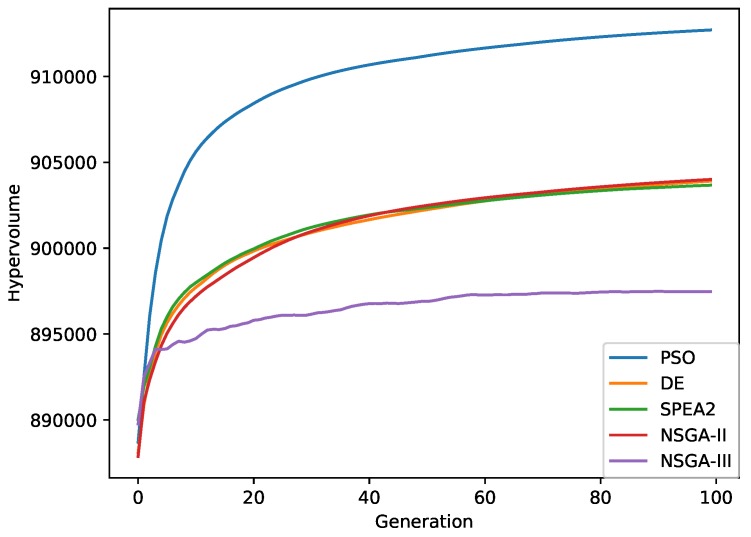
Averaged hypervolume metric over 100 generations for all multi-objective optimisation metaheuristics.

Figure 9 shows the average value of the HV performance metric for all algorithms over 100 generations. Note that the HV metric can be reduced during the experiment (NSGA-III algorithm). This is because neither multi-objective optimisation metaheuristic optimises the HV metric directly, unlike other search heuristics such as Hype [102]. As stated in Section 3.6, the best results were achieved by the PSO algorithm after the tenth generation, because, in all likelihood, it maximises the number of elements of the Pareto optimal set and the spread of solutions found through the leader selection mechanism. In contrast, note that the worst results were obtained with the NSGA-III algorithm.

Statistical hypothesis tests are needed to validate the HV values of independent experiments. The Kruskal–Wallis test and the Friedman test were used to determine whether the results were produced with the same probability distribution, i.e., if the search heuristics produced statistically significant different results. As a reminder, a *p*-value provides information on the significance or otherwise of a statistical hypothesis test. A *p*-value less than 0.05 was considered statistically significant. The Kruskal–Wallis test and the Friedman test results were equal to $1.21\times 10^{-7}$ and $2.14\times 10^{-6}$, respectively. Significant differences therefore existed between the results of all the algorithms.

Although the results were not the same, it cannot be established which were different or similar to the others. The Mann–Whitney–Wilcoxon U test was, therefore, applied in a pairwise manner, to determine whether the results of any one algorithm were significantly better than those of the other. Table 2 shows the pairwise results. As can be observed, there are significant differences between all the algorithms, except between NSGA-II and SPEA2 (p=0.0963) and between NSGA-II and DE (p=0.4497), which was probably due to the use of the NSGA-II algorithm as the selection algorithm on the truncation process of DE. However, these *p*-values are not appropriate for multiple pairwise comparisons, because they do not consider the other comparisons. Adjusted *p*-values (APVs) were therefore applied to solve that problem, which can be used to consider the results of multiple tests. In this study, the Bonferroni–Dunn procedure, the Hochberg procedure and the Hommel procedure were applied to adjust the *p*-values. Table 3 shows the adjusted *p*-values for multiple comparisons between all the metaheuristics. The adjusted *p*-values demonstrated that there were significant differences between the different groups, except in the comparisons between certain metaheuristics: SPEA2 vs NSGA-II and NSGA-II vs. DE.

**Figure 10:**
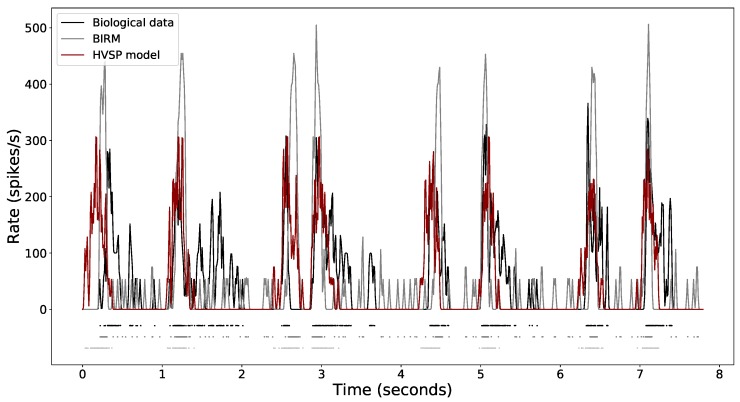
Spike rasters and normalised PSTHs of one retinal ganglion cell responding to 4 repeated trials of the same selected stimulus: biological data (black), tuned bioinspired retinal model (BIRM) (grey) and the Poisson spike generator (HVSP) model (red).

Last, Figure 10 shows the spike rasters of one retinal ganglion cell responding to four repeated trials of the same stimulus (top black row) compared to the predictions of the tuned retinal model under study—BIRM (middle grey row)—and the HVSP model (bottom red row), to show how the models predict retinal responses. A variety of parameters for the HVSP model were explored, based on the recommendations of the original study [49]. In the same graph, the peristimulus time histograms of the spike rasters are shown, which makes it possible to summarise the time-varying firing rate exhibited by the biological data and both models. PSTHs were computed by binning each response, then summing and filtering with a Gaussian function. Each set of raster data is represented together with the accumulated raster which gathers all repetitions for biological (black) and predicted cases (grey for the tuned BIRM and red for the HVSP model). In the case of the data predicted with the tuned model, the four rasters were generated by one of the individuals from the PSO Pareto front after performing an analysis and selection of the elements of the Pareto front. Rasters of RGC responses and the corresponding simulated responses from both models illustrate that the tuned model captures the structure of the RGC spike trains in a similar way to the HVSP model. The average firing rates of biological data, HVSP model output and tuned BIRM output were compared for calculation of the Pearson correlation coefficient (PCC) as a performance measure, with the purpose of quantifying the similarity between predicted and recorded firing rate traces. Both models can predicate the RGC response with PCC up to 0.45, consistent with previous results [103]. In addition, as can be seen in the figure, the adjusted BIRM is able to capture the regions with low firing rates with greater precision than the HVSP model (flat regions).

## 5. Conclusions

The tuning of a highly parameterisable retinal model has been presented in a novel contribution for the prediction of retinal ganglion cell responses by means of a multi-objective optimisation strategy. The contributions of this work are as follows. First, a generic automatic optimisation framework has been developed for accurate prediction of retinal responses produced by a real-time mathematical model of the retina that mimics vertebrate retinal behaviour. The design follows the principles of clean architecture, so this framework can easily be extended to the automatic adjustment of other retinal models, as well as adjusted to incorporate new metaheuristics. Second, the accuracy of the retinal model under evaluation has been compared by means of biological recordings from mice through four metrics, thereby achieving models of greater accuracy. Having conducted the initial experiments, we found that better results were obtained using all the metrics, instead of reducing the problem to a single objective. Last, five different search heuristics have been proposed to conduct the fine-tuning of the parameters of a bioinspired retinal model, with the hypervolume selected as the best comparative metric. For this particular multi-objective optimisation problem, the best results were achieved with the PSO algorithm, in so far as it produced the largest hypervolume, a large number of elements on the Pareto front and a good spread of solutions (with uniform and smooth vector distributions). Furthermore, a rigorous comparison between all the algorithms has been conducted by means of nonparametric statistical tests providing an accurate optimisation strategy. Adjusted models with the PSO algorithm predicted RGC responses with a good correlation, capturing the regions with low firing rates better than the HVSP model. Moreover, the adjusted BIRM can work in real-time, which the HVSP model cannot perform, which is desirable for the development of visual neuroprostheses. In conclusion, despite the obvious limitations of using an animal model that cannot be easily transposed to restore functional human vision, this work may serve as the basis for future experiments, to demonstrate the feasibility of bioinspired sensors and neuroprosthetic interfaces with the occipital cortex.

## Figures and Tables

**Table 1:** Chromosome parameters with their variation range.

Parameter	Range	Data Type
K	3–13	int
Leakage	10.0–15.0	float
Threshold	225.0–275.0	float
Persistence Time	3–7	int
Refractory Period	1.0–10.0	float
Frequency Modulator Factor	0.25–0.40	float

**Table 2:** Pairwise Mann–Whitney–Wilcoxon U test. Significant values are those *p*-values (<0.05) shown in bold.

	SPEA2	NSGA-II	NSGA-III	PSO	DE
SPEA2	-	0.0963	**0.0015**	**0.0002**	**0.0233**
NSGA-II	-	-	**0.0003**	**0.0002**	0.4497
NSGA-III	-	-	-	**0.0002**	**0.0002**
PSO	-	-	-	-	**0.0002**
DE	-	-	-	-	-

**Table 3:** Adjusted *p*-values for multiple comparisons between all metaheuristics. Significant values are those *p*-values shown in bold.

Comparison	Unadjusted	Bonferroni	Hochberg	Hommel
SPEA2 versus NSGA-II	0.0963	1.0000	0.1070	0.1070
SPEA2 versus NSGA-III	**0.0015**	**0.0300**	**0.0021**	**0.0021**
SPEA2 versus PSO	**0.0002**	**0.0042**	**0.0004**	**0.0004**
SPEA2 versus DE	**0.0233**	0.4668	**0.0292**	**0.0292**
NSGA-II versus NSGA-III	**0.0003**	**0.0057**	**0.0005**	**0.0005**
NSGA-II versus PSO	**0.0002**	**0.0031**	**0.0004**	**0.0004**
NSGA-II versus DE	0.4497	1.0000	0.4497	0.4497
NSGA-III versus PSO	**0.0002**	**0.0031**	**0.0004**	**0.0004**
NSGA-III versus DE	**0.0002**	**0.0042**	**0.0004**	**0.0004**
PSO versus DE	**0.0002**	**0.0031**	**0.0004**	**0.0004**
